# Biliary epithelium and liver B cells exposed to bacteria activate intrahepatic MAIT cells through MR1

**DOI:** 10.1016/j.jhep.2015.12.017

**Published:** 2016-05

**Authors:** Hannah C. Jeffery, Bonnie van Wilgenburg, Ayako Kurioka, Krishan Parekh, Kathryn Stirling, Sheree Roberts, Emma E. Dutton, Stuart Hunter, Daniel Geh, Manjit K. Braitch, Jeremy Rajanayagam, Tariq Iqbal, Thomas Pinkney, Rachel Brown, David R. Withers, David H. Adams, Paul Klenerman, Ye H. Oo

**Affiliations:** 1 Centre for Liver Research and NIHR Biomedical Research Unit in Liver Disease, Institute of Immunology & Immunotherapy, University of Birmingham, United Kingdom; 2 Peter Medawar Building for Pathogen Research, University of Oxford, United Kingdom; 3 Institute of Immunology and Immunotherapy, University of Birmingham, United Kingdom; 4 University Hospital of Birmingham NHS Foundation Trust, United Kingdom

**Keywords:** Human liver, Mucosal-associated invariant T cells, Biliary epithelium, *E. coli*, Immune response, Biliary firewall

## Abstract

**Background & Aims:**

Mucosal-Associated Invariant T (MAIT) cells are innate-like T cells characterised by the invariant TCR-chain, Vα7.2-Jα33, and are restricted by MR1, which presents bacterial vitamin B metabolites. They are important for antibacterial immunity at mucosal sites; however, detailed characteristics of liver-infiltrating MAIT (LI-MAIT) and their role in biliary immune surveillance remain unexplored.

**Methods:**

The phenotype and intrahepatic localisation of human LI-MAIT cells was examined in diseased and normal livers. MAIT cell activation in response to *E. coli*-exposed macrophages, biliary epithelial cells (BEC) and liver B cells was assessed with/without anti-MR1.

**Results:**

Intrahepatic MAIT cells predominantly localised to bile ducts in the portal tracts. Consistent with this distribution, they expressed biliary tropic chemokine receptors CCR6, CXCR6, and integrin αEβ7. LI-MAIT cells were also present in the hepatic sinusoids and possessed tissue-homing chemokine receptor CXCR3 and integrins LFA-1 and VLA-4, suggesting their recruitment via hepatic sinusoids. LI-MAIT cells were enriched in the parenchyma of acute liver failure livers compared to chronic diseased livers. LI-MAIT cells had an activated, effector memory phenotype, expressed α4β7 and receptors for IL-12, IL-18, and IL-23. Importantly, in response to *E. coli*-exposed macrophages, liver B cells and BEC, MAIT cells upregulated IFN-γ and CD40 Ligand and degranulated in an MR1-dependent, cytokine-independent manner. In addition, diseased liver MAIT cells expressed T-bet and RORγt and the cytokines IFN-γ, TNF-α, and IL-17.

**Conclusions:**

Our findings provide the first evidence of an immune surveillance effector response for MAIT cells towards BEC in human liver; thus they could be manipulated for treatment of biliary disease in the future.

## Introduction

Mucosal-associated invariant T (MAIT) cells are a recently identified subset of T cells with an evolutionarily conserved invariant T cell antigen receptor (TCR) α-chain, composed of the invariant α-chain Vα7.2-Jα33/Jα20/Jα12 in humans and Vα19-Jα33 in mice [1], [2]. They are restricted to the CD161^++^ population and are abundant in human blood, the intestinal mucosa and mesenteric lymph nodes [3], [4], [5]. MAIT cells respond to antigen presented on the highly phylogenetically conserved major histocompatibility complex (MHC) class I-related molecule, MR1, which possesses a unique antigen-binding cleft for vitamin B metabolites from pathogenic and/or commensal bacteria, and distinguishes MAIT cells from peptide- or lipid-recognizing αβ T cells [1], [6], [7]. MAIT cells can be activated by a wide variety of bacterial strains *in vitro*, and importantly they are crucial in mucosal immune defence in bacterial infection [8], [9], [10]. They respond in an MR1-dependent manner to antigen presenting cells (APC) cultured with bacteria and can also be activated via IL-12 and IL-18 in a TCR-independent manner [11], [12]. MAIT cell frequencies have been reported to be lower in bacterially-infected patients’ blood [10], [13].

Both hepatic sinusoids and biliary epithelial cells (BEC) are crucial in first-line defence towards pathogens in both the steady and disease state as the human liver is continuously exposed to intestinally-derived antigens from portal venous blood and biliary reflux [14]. A recent study suggested that immune cells in the hepatic sinusoids function as a firewall to prevent the systemic spread of gut-derived pathogens that evade the mesenteric immune system [15]. The presence of MAIT cells has been reported in healthy human liver sinusoidal fluids [16], however, their role in mucosa defence at the bile ducts, which are continuous with the gut lumen and its microbes, and form the first-line protection against biliary pathogens, is still unexplored [17], [18]. BEC are known to express antigen presenting molecules and can activate lymphocytes [19]. A recent report indicated that MAIT cells could efficiently lyse epithelial cells of the HeLa cell line that are infected with bacteria [20]. Taken together, these findings indicate that MAIT cells are likely to be important contributors to the maintenance of steady state immunity and the pathogenesis of inflammatory and biliary liver diseases, especially in response to bacterial exposure. Thus, in the current study, we used primary human liver tissues, obtained from both normal and diseased explanted human livers, to investigate the localisation and phenotype of intrahepatic/liver-infiltrating MAIT (LI-MAIT) cells, as well as their functional response to bacterially-exposed biliary epithelial surfaces in inflammatory biliary liver diseases.

## Materials and methods

### Isolation of liver-infiltrating lymphocytes (LIL), peripheral blood lymphocytes (PBL), and BEC

Venous blood, collected in EDTA, was obtained from healthy donors, and patients with inflammatory and autoimmune liver diseases (primary sclerosing cholangitis (PSC), primary biliary cirrhosis (PBC)) and alcoholic liver disease (ALD). Explanted diseased liver tissue was obtained from patients who underwent liver transplantation for end-stage liver diseases including PSC, PBC, ALD, and non-alcoholic steatohepatitis (NASH) or for acute liver failure from seronegative (NonA NonB (NANB)) hepatitis. Non-diseased liver tissues were obtained from unused donor liver tissues. All samples were collected with appropriate patient consent and local research ethics committee approval (LREC ref. CA/5192, 06/Q2708/11). Human LIL, PBL, and BEC cells were isolated from fresh liver tissue and peripheral blood as described previously [21].

### Localisation of TCR Vα7.2-expressing cells

Human liver tissues were stained with purified anti-TCR Vα7.2 (50 μg/ml, 3C10, BioLegend) or IgG1 isotype control to detect the localisation of Vα7.2^+^ cells. See Supplementary material.

### Phenotyping of intrahepatic and peripheral blood MAIT cells

Liver-infiltrating and blood MAIT cells were phenotyped directly *ex vivo* for the expression of surface markers, transcription factors and intracellular cytokines. See Supplementary material.

### MAIT cell response to *E. coli*-exposed antigen presenting cells

APCs: blood monocyte-derived macrophages, THP1, liver B cells or BEC were incubated overnight with paraformaldehyde-fixed *Escherichia coli* (*E. coli*) (DH5α, Invitrogen) at 25, 20, 1000, or 1000 bacteria per cell respectively. CD8^+^ T cells isolated from blood using CD8 Microbeads (Miltenyi Biotec) or CD3^+^ T cells isolated from liver by flow sorting were cultured with the *E. coli-*exposed APCs, in the presence of anti-CD107a (Pe or PeCy5) and blocking antibodies against IL-12p40/70 (5 μg/ml, C8.6, eBioscience), IL-18 (5 μg/ml, 125-2H, MBL International, USA) and MR1 (10 μg/ml) [22] as indicated. In some assays, anti-CD40 Ligand (CD40L)-PeCy7 was added. MAIT cell intracellular and surface markers were stained and data were acquired on a MACSQuant (Miltenyi Biotec) or CyAN (Dako) flow cytometer and analyzed using FlowJo (Tree Star Inc.). Autologous liver-infiltrating B cells and T cells were cell sorted by a Moflo Astrios cell sorter (Beckman Coulter). Cells were labeled with anti-CD3-PeCy7 and anti-CD19-APCVio770 to identify CD3^+^ T cells and CD19^+^ B cells respectively. Blood macrophages were generated by culturing CD14^+^ monocytes, isolated from blood using CD14 microbeads (Mlltenyi Biotec), with 100 ng/ml M-CSF (R and D Systems) for 7–9 days.‬‬‬‬‬‬‬‬‬‬‬‬‬

### Statistical analysis

GraphPad Prism 5.0 software (GraphPad software, San Diego, CA, USA) was used for statistical analysis. Comparisons of two populations were by the Mann-Whitney *U* test or *t* test. Comparisons of more than one population were by Friedman’s test with Dunn’s Multiple Comparison post-hoc test or by one-way ANOVA with Bonferroni’s multiple comparison post hoc test as indicated in the figure legend. Statistical significance was defined as *p* value <0.05. Error bars on graphs are presented as median ± interquartile range or mean ± SEM. Values in text are given as median and overall range (in brackets).

## Results

### Intrahepatic MAIT cells preferentially reside in peri-biliary areas of portal tracts

**Fig. 1:**
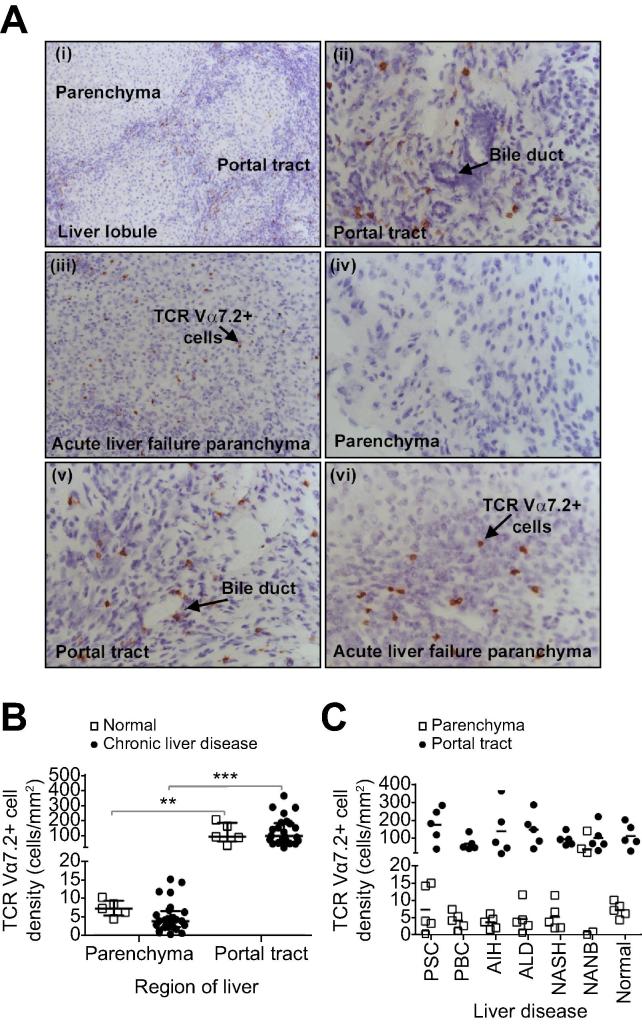
**Peri-biliary localisation of Vα7.2^+^ cells in chronic liver diseases.** (A) Representative staining for Vα7.2 on frozen liver sections viewed at 10× (i and iii) or 40× (ii, iv, v, and vi) magnification. Distribution of Vα7.2+ cells in the parenchyma (i and iv) and portal tract (i, ii, and v) in PSC and in the parenchyma (iii and vi) in seronegative acute liver failure. (B) Densities of Vα7.2+ cells in parenchyma and portal tracts of normal and chronically diseased livers (∗∗*p* <0.01; ∗∗∗*p* <0.0001 by Mann-Whitney *U* test). (C) Vα7.2+ cell density data according to diseases. Data are median ± interquartile range.

**Fig. 2:**
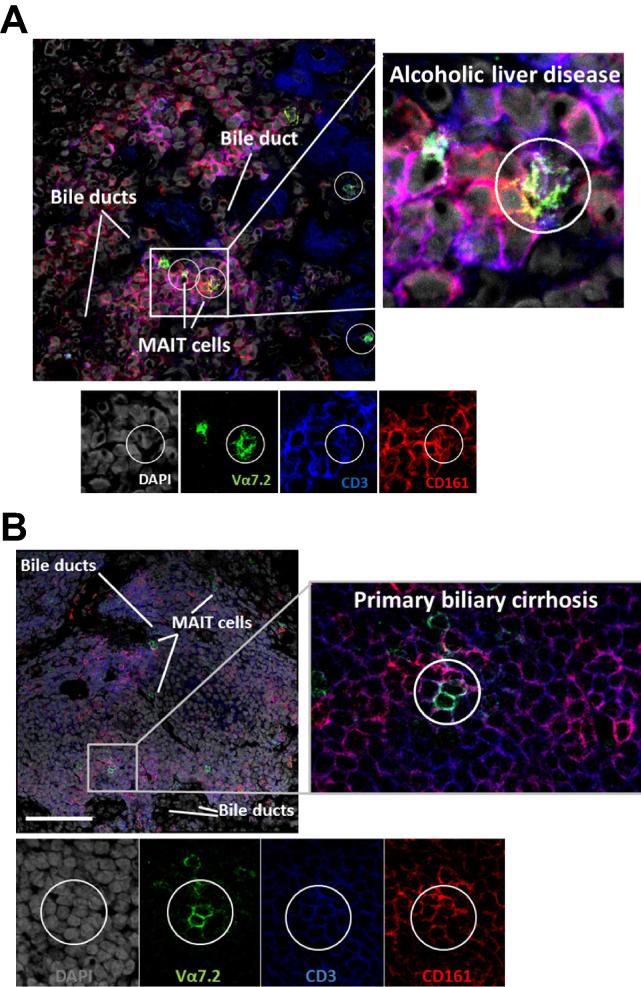
**CD3^+^CD161^+^Va7.2^+^ cells reside close to bile ducts in portal tracts.** Representative confocal immunofluorescence staining for CD3, CD161, and Va7.2 on frozen sections from explanted human livers diagnosed with Alcoholic liver disease (A) and Primary Biliary Cirrhosis (B). DAPI nuclear stain reveals liver architecture indicating sites of bile ducts. Images are representative of staining of four different diseased livers, scale bar shows 100 μm.

We examined the localisation of LI-MAIT cells in normal and diseased human livers by immunohistochemistry staining for TCR Vα7.2. Most Vα7.2^+^ cells resided around bile ducts in portal tracts with few detected in the parenchyma (Fig. 1A, B; Supplementary Fig. 2). The distribution was similar in normal, autoimmune, and non-autoimmune diseased livers (Fig. 1C; Supplementary Fig. 2) similar to other immune subsets (Supplementary Fig. 1). Interestingly, in acute, seronegative liver failure, increased infiltration of Vα7.2^+^ cells to the parenchyma was noted (Fig. 1A iii, vi, 1C; Supplementary Fig. 3) when compared to normal livers or any of the chronic liver diseases studied (Fig. 1A i, iv). The overall frequency of Vα7.2^+^ cells appeared increased in PSC compared to the other liver diseases (Fig. 1C). By flow cytometry, we showed that the majority of Vα7.2^+^ lymphocytes in normal livers (63.6% (24.4–93.2%)) and over one-third in diseased (40.5% (11.6–75.2%)) were CD3^+^CD161^++^ MAIT cells (Supplementary Fig. 4). We confirmed the predominant localisation of CD3^+^ CD161^+^ Vα7.2^+^ MAIT cells in peri-biliary regions of portal tracts by both immunohistochemistry (Fig. 1Aii, v; 1C) and confocal microscopy (Fig. 2).

### Frequencies of MAIT cells are reduced in liver diseases, with an increase in the CD4^+^ MAIT cells

**Fig. 3:**
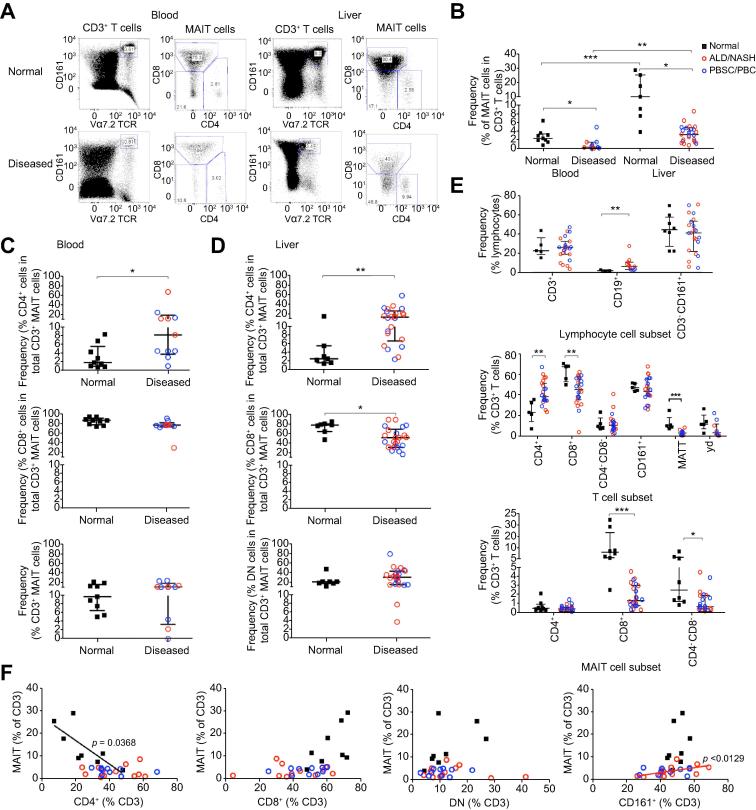
**Flow cytometric analysis of MAIT cell frequencies in chronic liver disease and their correlations with other immune subsets.** Representative FACS plots (A) and summary frequency data for total CD3^+^ MAIT (B) and CD4^+^, CD8^+^ and CD4^−^ CD8^−^ (DN) MAIT subsets in normal and diseased blood (C) and liver (D). (E) Frequencies of intrahepatic MAIT and other immune cells. (F) Correlation of CD3^+^ MAIT cell frequencies with total CD4^+^, CD8^+^, DN, and CD161^+^ T cell frequencies in normal and diseased livers. Data are median ± interquartile range. ∗*p* <0.05; ∗∗*p* <0.01; ∗∗∗*p* <0.001 by Mann-Whitney *U* test (B–E) or Spearman’s rank correlation (F).

Next, using flow cytometry we compared frequencies of CD3^+^ CD161^++^ Vα7.2^+^ MAIT cells in intrahepatic liver infiltrates and in blood from normal and diseased tissues. Increased frequency of MAIT cells in liver compared to blood was observed in both normal and diseased states (Fig. 3A, B). The frequency of liver and blood MAIT cells in total CD3^+^ T cells was decreased in chronic liver diseases (Fig. 3A, B). In liver as in blood, CD8^+^ cells represented the major MAIT cell subset (Fig. 3C, D). However, in disease, the proportion of CD4^+^ MAIT cells was significantly increased in both the blood and liver, which in liver, was compensated for by a significant reduction in the CD8^+^ MAIT cell frequency (Fig. 3C, D). MAIT cells were unique among the T cell subsets that we examined in showing a reduced frequency with disease (Fig. 3E). We observed a negative correlation between total MAIT cells and total CD4^+^ T cells in normal livers but found no sign of this correlation in disease. Conversely there was a trend towards a positive correlation of MAIT cells with CD8^+^ T cells in normal livers. In non-autoimmune livers we noticed a positive correlation with CD161^+^ T cells. No relationships were found between MAIT cells and CD4^−^CD8^−^ double negative (DN) T cells in either normal or diseased livers (Fig. 3F). In disease, the proportion of CD4^+^ cells within the MAIT cell population was approximately 2-fold greater than that occurring for total CD4^+^ cells within the total T cell population; however the frequency of CD4^+^ MAIT cells among total T cells did not alter with disease, rather the CD8^+^ and DN MAIT cell frequencies among total T cells decreased significantly in disease, accounting for the rise in the proportion of CD4^+^ cells within the MAIT cell population (Fig. 3E).

### Tissue-homing chemokine receptor, integrin and cytokine receptor expressions of intrahepatic MAIT cells

**Fig. 4:**
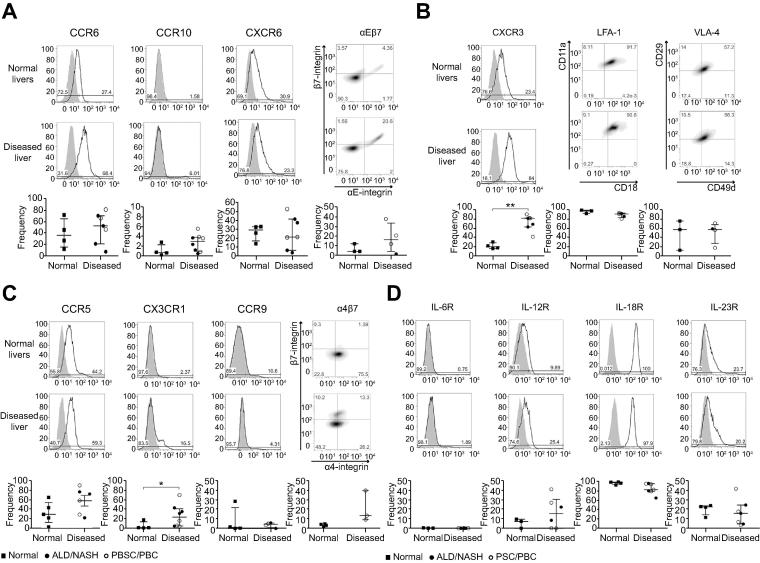
**Expressions of tissue-homing chemokine, integrin and cytokine receptors on intrahepatic MAIT cells in normal and chronic liver diseases.** Chemokine receptor and integrin expression profiles (A, B, and C) and cytokine receptor profiles (D) were determined by flow cytometry gating on the total CD3^+^ MAIT cell population. Representative overlays for marker (line) and isotype control (grey shading) and total summary data are shown. Summary data are median ± interquartile range. ∗*p* <0.05; ∗∗*p* <0.01; ∗∗∗*p* <0.001 by Mann-Whitney *U* test.

Chemokine receptors, CXCR6 and CCR6 and integrin αEβ7 have been implicated in lymphocyte recruitment to biliary epithelium [21], [23], [24]. All three were expressed by LI-MAIT cells from both diseased and normal livers [CXCR6: (normal: 29% (14–33%); diseased: 22% (4–52%)), CCR6 (normal: 36% (12–72%); diseased: 53% (7–81%)), αEβ7 (normal: 4% (4–12%); diseased: 16% (2–37%))] (Fig. 4A).

Sinusoidal recruitment to inflamed tissue involves the chemokine receptor CXCR3, which responds to interferon-dependent ligands, CXCL9/10/11 in inflamed tissues, and the integrins LFA-1 and VLA-4 [25]. Almost all LI-MAIT cells expressed LFA-1 (normal: 96% (92–98%); diseased: 91% (81–93%)) and most expressed VLA-4 (normal: 57% (13–75%); diseased: 58% (19–71%)) (Fig. 4B). LI-MAIT cells from diseased livers showed significant upregulation of CXCR3 compared to LI-MAIT cells from normal tissue (normal: 21% (17–31%); diseased: 81% (42–89%), *p* <0.01) (Fig. 4B). Increased expression of CX3CR1 and CCR5 was also noticed in disease (Fig. 4C). Since MAIT cells are believed to expand in the gut in response to bacterial antigens, we screened for expression of the gut-homing chemokine receptor CCR9 and integrin α4β7 [26] but detected little expression on LI-MAIT cells from either normal or diseased tissue (Fig. 4C). Given the change in the balance of CD4^+^/CD8^+^/DN^+^ MAIT cells in diseased livers we assessed whether there were any subset-specific differences in homing receptor expression profile. We observed little difference in percentage and intensity of expression across subsets in their expression of CCR6, CCR5 and CX3CR1. Interestingly, however, higher frequencies of CD4^+^ MAIT expressed CXCR3 compared to CD8^+^ or DN MAIT in normal livers, although the intensity of expression did not differ across subsets. In disease, frequencies of CD4^+^ and CD8^+^ CXCR3-expressing cells did not differ but the intensity of expression on CD4^+^ cells was significantly greater than on CD8^+^ or DN cells (Supplementary Fig. 5).

We also evaluated the expression of cytokine receptors whose cognate cytokines are known to be secreted by liver-resident cells and to mediate MAIT cell activation *in vitro* [12]. The pattern of cytokine receptor expression was similar in diseased and non-diseased liver tissue. IL-18R was highly expressed (normal: 97% (93–99%); diseased: 83% (65–96%)). There was low-to-moderate expression of IL-12R (normal: 7.5%; diseased: 15.7%) and IL-23R (normal: 23%; diseased: 16%). IL-6R was not detected on LI-MAIT cells (Fig. 4D).

### Phenotypic characterisation of liver-infiltrating MAIT cells

There was no overlap of the LI-MAIT cell population with γδ T cells (Supplementary Fig. 6A). Although LI-MAIT cells had moderate expression of the Natural Killer (NK) cell marker CD56, they had low expression of other NK cell phenotypic markers such as NKG2D and NKp46 (Supplementary Fig. 6B). LI-MAIT cells were mostly CD45RA^−^CCR7^−^ effector memory (Supplementary Fig. 7A) and expressed the activation marker CD69 but lacked CD40L (Supplementary Fig. 7B). LI-MAIT cells showed ubiquitous surface expression of CD95 (93%) but lacked CD95L (Supplementary Fig. 7C). They were also found to have constitutive expression of CD26 and to express the adenosine-pathway receptors CD39, and CD73 (Supplementary Fig. 8A).

### Diseased liver-infiltrating MAIT cells produce IFN-γ, TNF-α, IL-17, and granzyme B

**Fig. 5:**
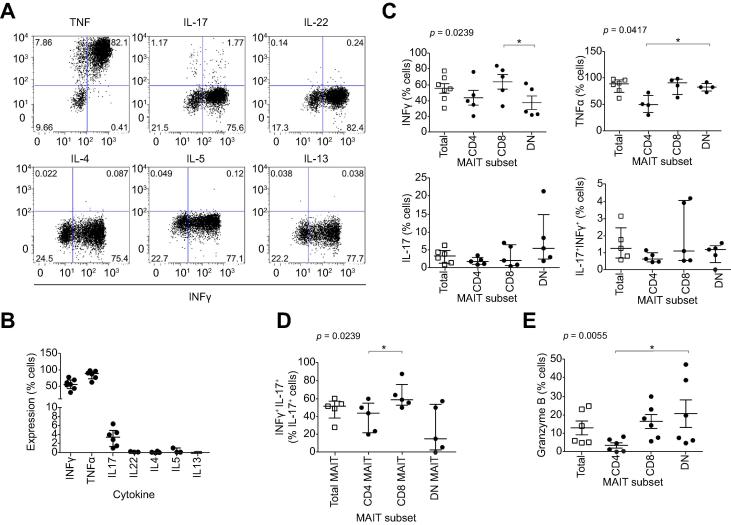
**Cytokine and cytolytic factor expression by liver-infiltrating MAIT cells.** Intrahepatic MAIT cell production of Th1 (IFN-γ, TNF-α), Th2 (IL-4, IL-5, IL-13) and Th17 (IL-17, IL-22) cytokines was examined by flow cytometry. Representative dot-plots for each cytokine *vs.* IFN-γ, expression (A) and summary data by MAIT cells (B) and MAIT cell subsets (C and D) are shown. Summary data for *ex vivo* granzyme B expression in total MAIT and MAIT cell subsets (E). Data are median ± interquartile range. ∗*p* <0.05; ∗∗*p* <0.01; by Dunn’s post hoc test following Friedman’s test.

To determine possible effector functions of LI-MAIT cells, we examined the cytokines and cytotoxic granzyme produced by MAIT cells *ex vivo*. LI-MAIT cells showed high frequencies of IFN-γ (55%) and TNF-α expression (89%) and low frequencies of IL-17 production (3.5%) but IL-22 and Th2-cytokines including IL-4, IL-5, and IL-13 were barely detected (Fig. 5A, B). We noticed that approximately 50% of IL-17-producing cells had a dual Th1/Th17 phenotype, secreting IFN-γ. Consistent with their ability to produce both Th1 and Th17-type cytokines, LI-MAIT cells expressed the transcription factors T-bet and RORc, (Supplementary Fig. 8B). Examining *ex vivo* stores of cytotoxicity factor, we found moderate frequencies of granzyme B-expressing LI-MAIT cells (10% (5–25%)) (Fig. 5F).

### Activation of blood and liver-infiltrating MAIT cells by antigen presenting cells in an MR1 dependent manner

**Fig. 6:**
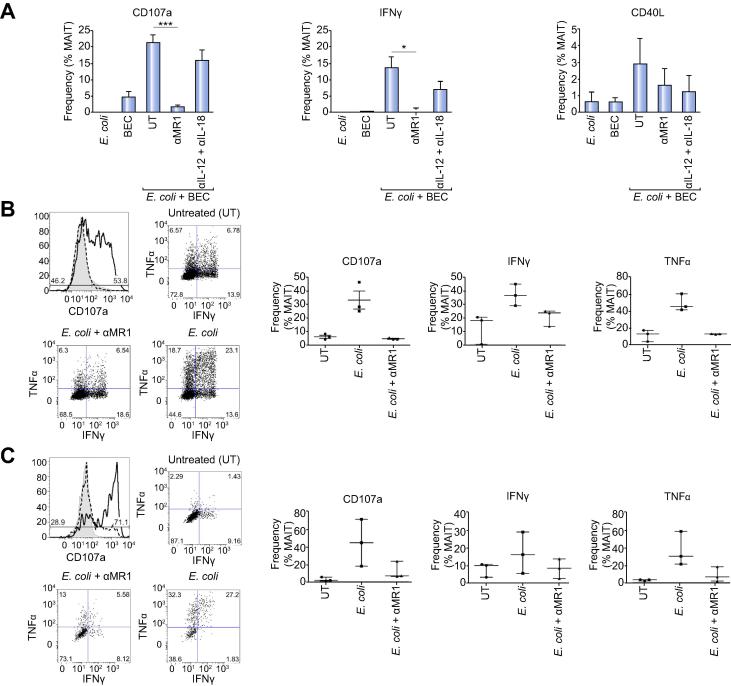
**MR1-dependent activation of blood and liver MAIT cells by professional and non-professional antigen presenting cells (APC) exposed to bacteria.** APC exposed to *E. coli* were co-cultured with MAIT cells and their activation examined by flow cytometry for CD107a, IFN-γ, TNF-α, CD40L. Dependency on MR1 or IL-12 and IL-18 was assessed by antibody blocking. Activation of blood MAIT by blood macrophages (A) and activation of liver MAIT by THP1 cells (B) and autologous intrahepatic B cells (C) were analyzed. Data are mean ± SEM (A) and median ± interquartile range (B and C). In overlays: untreated (shaded); *E. coli* (solid line); *E. coli* + αMR1 (dotted line). ∗*p* <0.05; ∗∗∗*p* <0.001 by paired *t* test. UT = untreated.

MAIT cells can be activated in response to bacterial metabolites in an MR1- and/or IL-12/IL-18-dependent manner by professional and non-professional APC, such as HeLa cells, B cells and THP1 cells [20], [27]. As a model to study activation by macrophages, we exposed blood MAIT cells to monocyte-derived macrophages that had been treated with or without *E. coli* and examined the expression of the degranulation marker CD107a, IFN-γ, and CD40L. MAIT cells expressed CD107a and IFN-γ in an MR1-dependent manner. They also showed a tendency for CD40L expression (Fig. 6A). These responses were MR1-dependent but independent of IL-12 or IL-18. Both THP1 cells (Fig. 6B) and liver-infiltrating B cells (Fig. 6C), pre-treated with *E. coli*, activated LI-MAIT cells from diseased livers by inducing the expression of CD107a, IFN-γ and TNF-α in an MR1-dependent manner.

### Bacterially-exposed primary human biliary epithelial cells activate MAIT cells in an MR1-dependent, cytokine-independent manner

**Fig. 7:**
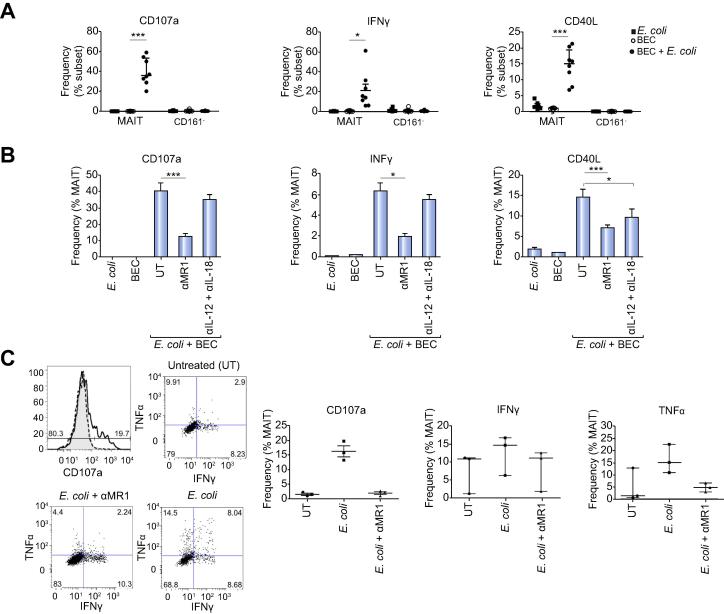
**Bacterially-exposed primary human biliary epithelial cells activate both blood and liver MAIT cells in an MR1 dependent manner.** Biliary epithelial cells exposed to *E. coli* were co-cultured with sorted blood CD8^+^ (A and B) or liver CD3^+^ (C) T cells and activation of CD161^++^ Va7.2^+^ MAIT cells examined by flow cytometry for CD107a, IFNγ-, TNFα, CD40L. Dependency on MR1 or cytokines IL-12 and IL-18 was assessed using function-blocking antibodies. Data are mean ± SEM (A and B) and median ± interquartile range (C). In overlays: untreated (shaded); *E. coli* (solid line); *E. coli* + αMR1 (dotted line). ∗*p* <0.05; ∗∗∗*p* <0.001 by t test. UT = untreated.

Since we observed LI-MAIT cells around bile ducts in the portal tracts we proceeded to examine whether MAIT cells may respond to bacterial infection associated with the biliary epithelium. We therefore co-cultured blood derived MAIT cells with primary human BEC with or without *E. coli* and observed selective activation by the MAIT population in the presence of BEC presenting *E. coli*. The Vα7.2^+^CD161^−^ cells within the same culture did not respond (Fig. 7A). Increased expression of CD107a and IFN-γ was MR1-dependent, however, blocking the cytokines IL-12 and IL-18 did not have any effect (Fig. 7B). CD40L upregulation was significantly inhibited by blocking either MR1 or the cytokines IL-12 and IL18 (Fig. 7B). We performed the same assay with T cells isolated from diseased livers. LI-MAIT responded to BEC presenting *E. coli*, upregulating CD107a, IFN-γ and TNF-α in an MR1-dependent manner (Fig. 7C).

## Discussion

Although MAIT cells have recently been reported in normal human liver perfusate, detailed characteristics of liver-infiltrating MAIT cells in normal and diseased state, and their role in biliary epithelial mucosa protection remained unexplored [3], [16], [28], [29]. The biliary epithelium is in continuity with the intestinal gut flora and thus provides another potential portal of pathogen entry from the gut to the liver. As such, resident immune cells including MAIT cells that localised around bile ducts in the portal tracts play an important role in protection against invading bacteria. MAIT cells respond to microbial antigens to provide protection at epithelial and mucosal surfaces. The liver regulates tolerance to food antigens and at the same time acts as a firewall to prevent intestinal microbes entering the systemic circulation [15]. We observed the overall MAIT cell frequency to be significantly higher in the normal liver compared to diseased liver, which differed from conventional CD4^+^, CD8^+^, or DN T cells. This suggests that MAIT cells are a major class of T cell recruited to the normal liver in order to act as a firewall and protect the biliary epithelium, therefore playing an important role in immune surveillance and homeostasis at the biliary mucosal barrier.

Immunohistochemical and confocal fluorescence staining of human liver tissue demonstrated that intrahepatic MAIT cells are present in the sinusoids. During an immune response to invading infection (e.g. bacterial infection) or during an inflammatory reaction, lymphocytes are recruited to liver tissue in response to combinations of locally expressed chemokines [14]. Hepatic inflammation leads to upregulation of IFN-inducible chemokines CXCL9/10/11, ligands for CXCR3, along with increased expression of the adhesion molecules ICAM-1 and VCAM-1 on inflamed human liver sinusoids [25], [30], [31]. We detected a significantly higher level of the chemokine receptor, CXCR3, on intrahepatic MAIT cells in the diseased state, as well as the presence of integrins LFA-1 and VLA-4. VLA-4 is essential in CD8^+^ MAIT cell infiltration to central nervous system in multiple sclerosis [32]. Thus, these molecules are involved in MAIT cell recruitment from blood. Intrahepatic Vα7.2^+^ CD161^++^ MAIT cells were highly enriched for IL-18Rα expression in the livers, which would allow the cells to respond to high local levels of IL-18 in the inflamed hepatic microenvironment [12], [33] and to interact with IL-18 secreted by Kupffer cells, which we observed to reside in the hepatic sinusoid [15], [34].

We observed enrichment of MAIT cells in the liver compared to in blood by flow cytometry and also demonstrated by immunohistochemistry and confocal microscopy that they were concentrated preferentially in portal tracts, where the majority of CD3^+^ CD161^+^ Vα7.2^+^ cells localised in the peri-biliary regions, often in close contact with bile ducts. BEC in their normal state secrete the chemokine CCL20 and express the cell adhesion molecule E-cadherin, both of which are enhanced in the diseased state [21]. Intrahepatic MAIT cells in either normal or diseased state expressed the chemokine receptors CCR6 and CXCR6, and the E-cadherin receptor, integrin αEβ7, which would allow them to migrate to the peri-biliary region in response to CCL20 and CXCL16 secreted by BEC [21], [23], [35]. We propose that CCR6^+^, CXCR6^+^ and αEβ7-expressing intrahepatic MAIT cells are retained close to the bile ducts in steady state to provide protection against ascending bacterial infection from the gut [23], [24], [36], [37]. VLA-4 on intrahepatic MAIT cells would also interact with VCAM-1 on the bile ducts, an interaction known to provide survival signals for lymphocytes [38]. Thus, activated, effector memory MAIT cells in the human liver are ready to protect the biliary mucosa in both the steady and diseased state [39], [40].

A higher prevalence of intrahepatic MAIT cells around bile ducts was apparent by immunohistochemistry in PSC compared to other chronic liver diseases, including other biliary diseases such as PBC. This is an interesting observation as PSC is a biliary disease driven by mucosal T cells and associated with recurrent ascending infections [41]. Surprisingly, although MAIT cells are found in the gut and associated with inflammatory bowel disease and colonic cancer [29], [40], we detected very few cells that expressed the gut-homing integrin α4β7 or chemokine receptor CCR9, suggesting that hepatic MAIT cells are most likely not derived from the gut.

Increased frequency of MAIT cells in the hepatic parenchyma of patients with severe seronegative acute liver failure was also noted with immunohistochemistry. Seronegative hepatitis is characterised by a progressive, marked hepatocyte-necrosis leading to acute liver failure and is associated with bacterial translocation [42]. Therefore, in this scenario, the effector functions of MAIT cells might contribute to acute liver injury. Intrahepatic cells not only expressed CD26 constitutively [39], [43], but also expressed CD39 and/or CD73, two ectoenzymes involved in immune regulation via generation of immunosuppressive adenosine [44]. Thus, hepatic MAIT cells include cells with both effector and regulatory functions which may confer either proinflammatory or immune-regulatory properties depending on the context and timing of the hepatic inflammation.

There needs to be local protection against intestinal pathogens at the biliary epithelium due to its continuity with the gut flora where both commensal and pathogenic bacteria reside [45]. Our data suggest that MAIT cells could play an important role in this. The recognition of antigen by MAIT cells is mediated via the MR1 molecule, which can present microbial vitamin B-derived compounds in its antigen-binding cleft [7]. MR1 is expressed on APC such as B cells [27]. We noted close localisation of B cells around bile ducts, and they mediated the activation of diseased liver-infiltrating MAIT cells in the presence of *E. coli*. Human BEC can also act as non-professional APC by expressing MHC and co-stimulatory molecules under some circumstances [19], [46]. We found that indeed, both LI-MAIT and blood MAIT cells co-cultured with BEC exposed to *E. coli* degranulated and secreted IFN-γ. Importantly, this mechanism was MR1-dependent but independent of IL-12 and IL-18 cytokines, despite the presence of these receptors on intrahepatic MAIT cells. This finding suggests that MAIT cells will only be fully activated in the presence of bacteria that have breached the epithelial barrier. Intrahepatic MAIT cells also secreted IL-17 and therefore likely possess not only an antibacterial function but also mucosa-regeneration properties similar to other Th17 cells [21], [47].

CD40L upregulation was also observed on MAIT cells in response to bacterial presentation by BEC. CD40L on immune cells such as lymphocytes and macrophages can induce BEC apoptosis via epithelial CD40 [33], [48], [49]. CD40L upregulation was mediated both by *E. coli* presented by MR1 as well as IL-12 and IL-18, providing a mechanism through which MAIT cells could drive bile duct damage in inflammatory liver disease in the absence of infection, in a non-specific manner. Intrahepatic CD4^+^, CD8^+^, and DN MAIT cells also secreted TNF-α and IFN-γ, which would be expected to contribute to their proinflammatory functional activity, and expressed granzyme B, which is crucial for cytolytic activity [11], [39], [50], [51].

Taken together, our findings provide the first evidence that intrahepatic MAIT cells in the human liver can respond to bacterial antigens presented by the biliary epithelium, B cells or macrophages by expressing IFN-γ, TNF-α, CD40L, and degranulating, and they have the capacity to secrete IL-17 upon activation. This suggests that intrahepatic MAIT cells play an important part in the biliary firewall that prevents bacteria from the gut entering the normal liver and then the systemic circulation via the bile ducts. We therefore propose that intrahepatic MAIT cells act as guardians in biliary mucosa protection at steady normal state. Whether they play a role in the pathogenesis of inflammatory liver disease requires further study.

## Financial support

Dr Ye Htun Oo was funded by Clinician Scientist Award from the 10.13039/501100000265Medical Research Council (G1002552), United Kingdom; Queen Elizabeth Hospital Charity, United Kingdom; NIHR Biomedical Research Unit in liver disease Birmingham, United Kingdom and 10.13039/501100000863Bowel Disease Research Foundation Grant, United Kingdom.

Professor Paul Klenerman was funded by the 10.13039/100004440Wellcome Trust (WT 091663MA), United Kingdom, NIHR Biomedical Research Centre Oxford, NIHR Senior Investigator award, Oxford Martin School, United Kingdom. MRC STOP-HCV consortium, United Kingdom and NIH NIAID 1U19AI082630-02, USA.

## Conflict of interest

The authors who have taken part in this study declared that they do not have anything to disclose regarding funding or conflict of interest with respect to this manuscript.

## Authors’ contributions

HJ, YO, BW, AK, and PK designed the study. HJ, BW, AK, KP, KS, SR, ED, SH, DG and MB collected and analysed the data. RB and DW advised on histological staining and analysis. JR, TP and TI contributed samples collection and intellectual input. HJ and YO drafted and wrote the manuscript. HJ, BW, AK, DA, PK and YO revised the manuscript critically for intellectual content. All authors gave intellectual input to the study and approved the final version of the manuscript.
