# Supplementary material for: Biliary epithelium and liver B cells exposed to bacteria activate intrahepatic MAIT cells through MR1
Source: J Hepatol. 2016 May;64(5):1118–27. doi: 10.1016/j.jhep.2015.12.017 (PMC4822535; doi:10.1016/j.jhep.2015.12.017)
Supplement: Supplementary data — Supplementary methods and Supplementary Figs. 1–8. [file mmc1.pdf]

Biliary epithelium and liver B cells exposed to bacteria activate intrahepatic MAIT cells through MR1

Hannah C Jeffery, Bonnie van Wilgenburg, Ayako Kurioka, Krishan Parekh, Kathryn Stirling, Sheree Roberts, Emma E Dutton, Stuart Hunter, Daniel Geh, Manjit K Braitch, Jeremy Rajanayagam, Tariq Iqbal, Thomas Pinkney, Rachel Brown, David R Withers, David H Adams, Paul Klenerman and Ye Htun Oo

Table of contents

Supplementary methods……………………………………………………..2

Supplementary Fig 1…………………………………………………………..8

Supplementary Fig 2…………………………………………………………..9

Supplementary Fig 3…………………………………………………………10

Supplementary Fig 4…………………………………………………………11

Supplementary Fig 5…………………………………………………………12

Supplementary Fig 6…………………………………………………………13

Supplementary Fig 7…………………………………………………………14

Supplementary Fig 8…………………………………………………………15

Supplementary methods

Isolation of liver infiltrating lymphocytes (LIL) and biliary epithelial cells (BEC)

Human LIL were isolated from fresh liver tissue as described previously[19]. Briefly, explanted liver tissue was diced into 5mm^3 cubes, washed with Phosphate Buffered Saline (PBS), and then homogenised in a Seward stomacher 400 circulator (260rpm, 5mins). The homogenate was filtered through fine (63 micron) mesh (John Staniar and Co, Manchester, UK) and the lymphocytes were isolated by density gradient separation using Lympholyte (VH Bio, Gateshead, UK) at 800 x *g* for 20minutes. The lymphocyte layer was collected and washed with PBS. Cell viability was assessed by trypan blue exclusion. Peripheral blood lymphocytes were likewise isolated from whole blood by density gradient separation using Lympholyte.

Primary BEC were isolated from explanted human diseased and normal livers as previously described[20]. Liver tissue underwent mechanical and enzymatic digestion with Collagenase type 1A (Sigma Aldrich, Dorset, UK) followed by density gradient centrifugation on 33%/77% Percoll (GE Healthcare, Buckinghamshire, UK). The cholangiocytes were purified by immunomagnetic isolation using antibodies against the cholangiocyte-specific receptor HEA 125 (Progen, Heidelberg, Germany) and then cultured to confluence in Dulbecco's Modified Eagle medium, Hams F12 (1:1) supplemented with 10%

heat-inactivated human serum, glutamine (2 mM, Gibco, California, USA), hepatocyte growth factor (10 ng/ml, Peprotech, London, UK), epidermal growth factor (10 ng/ml, Peprotech), hydrocortisone (2 µg/ml, Peprotech), cholera toxin (10 ng/ml, Sigma Aldrich), tri-iodo-thyronine (2 nM, Sigma Aldrich) and insulin (0.124 U/ml) with penicillin (100 IU/ml) and streptomycin (100 µg/ml, Gibco, California, USA). Culture flasks were coated with rat-tail collagen (Sigma Aldrich).

Localization of Vα7.2-expressing cells in human inflammatory liver diseases by immunohistochemistry staining on frozen human liver sections

Cubes of liver tissues (1-1.5cm^3) were cut, snap frozen in liquid nitrogen and stored at -80°C. Tissue was then embedded in Cryoembed (Leica Biosystems, Newcastle upon Tyne, UK) and 7μm thick sections cut using a cryostat with a specimen temperature of -13°C and chamber temperature of -20°C. The, air dried sections were put onto poly-L-lysine coated slides and after fixation for 5minutes in acetone, slides were air dried, wrapped in foil and stored at -20°C until staining. Thawed sections were fixed in acetone for 5minutes, washed twice with 0.1% Tween-PBS buffer (5minutes/wash) and endogenous peroxidase activity quenched by 20minutes incubation in 0.3% hydrogen peroxide (Sigma Aldrich) in methanol. After washing, the tissue was blocked for 20minutes with casein (1 in 10 in PBS) (Vector Laboratories, Burlingame, CA). This was followed, after washing, by incubation for 1hour in primary antibody (purified anti-TCR Vα7.2 (50μg/ml, 3C10, BioLegend) or

IgG1 isotype control) diluted in PBS. After washing, sections were covered with Impress secondary reagent (Vector Laboratories) for 30minutes at room temperature and following washing, sections were developed using ImmPACT^{TM} DAB reagent (Vector Laboratories) diluted as per the manufacturer's instructions. After 10minutes, excess DAB was removed by rinsing with distilled water. Sections were finally counterstained for 2minutes with filtered haematoxylin solution (Leica Biosystems). This was developed by incubation in cold water for 2.5minutes followed by hot water for 2.5minutes. Once dry, slides were mounted using DPX (Cellpath, Newtown Powys, UK) and imaged on a Zeiss Axioskop 40 Microscope with a x20 or x40 objective lens. Regions of parenchymal and portal tract tissue were identified and their areas measured using AxioVision SE64, Release 4.9 software. Numbers of TCR Va7.2$^+$ cells/mm^2 tissues were counted.

Localization of Vα7.2-expressing cells in human inflammatory liver diseases by immunofluorescence staining and confocal microscopy on frozen human liver sections

7μm thick sections of frozen human liver tissue were stained with mouse antibodies raised against the following human antigens: CD3 (clone UCHT1, 1:25, BioLegend), CD161 (clone HP-3G10, 1:50, BioLegend), and TCR Vα7.2 (clone 3C10, 1:10, BioLegend). Detection of Vα7.2 expression required amplification using goat anti-mouse-IgG-FITC (1:150, Southern Biotech), and then rabbit anti-FITC-AF488 (1:200, Life Technologies) and then with donkey anti-rabbit-IgG-AF488 (1:200, Life Technologies). Biotinylated anti-CD3 antibodies were detected with SA-AF555 (1:500, Life Technologies). Sections

were counterstained with DAPI (Invitrogen) and mounted using ProLong Gold (Invitrogen). Slides were analysed on a Zeiss 780 Zen microscope (Zeiss).

Phenotyping of freshly isolated intrahepatic and peripheral blood MAIT cells

MAIT cell phenotypes were examined by flow cytometry. In all protocols, dead LIL were identified by 30minute incubation, 4°C, with the e506 viability dye (eBioscience, Hatfield, UK) prior to staining with antibodies. To analyse expressions of surface antigens, cells were incubated for 30minutes on ice with antibodies against CD3, CD4, CD8, CD161, Vα7.2 and the antigen(s) of interest in 2% foetal bovine serum (FBS) (Sigma) diluted in PBS. After washing with 2% FBS, cells were fixed for 10minutes with 3% formaldehyde solution (Sigma Aldrich). To analyse expressions of intracellular transcription factors and cytotoxicity factors, cells were stained for surface markers CD161 and Vα7.2 (Biolegend) then fixed overnight at 4°C, permeabilised and stained using the Foxp3/Transcription factor staining set (eBioscience) with antibodies against CD3, CD4, and CD8 and the transcription factors Tbet and RORγt or cytotoxicity factor granzyme B. To analyse *ex vivo* cytokine production, LIL were plated in 24-well plates at density 2x10^6 cells/ml in RPMI supplemented with 10% human serum and rested overnight at 37°C. They were then stimulated with Phorbal Myristate Acetate (PMA) (25ng/ml, Sigma Aldrich) and ionomycin (1μM, Sigma Aldrich) for 5hours. Cytokine secretion was blocked by addition of Brefeldin A (5μg/ml, Sigma Aldrich) during the final 4hours. 4-6x10^6 cells were then transferred to each FACS tube for staining.

Cells were washed with PBS and dead cells stained using the e506 viability dye (eBiosciences) at 4°C for 30minutes. Afterwards cells were fixed, permeabilised and stained using the Foxp3/Transcription factor staining set (eBioscience) with antibodies against CD3 CD4, CD8, CD161 and Vα7.2 (Miltenyi Biotec) and with antibodies against cytokines IFN-γ, TNF-α, IL-17, IL-22, IL-4, IL-5 and IL-13 according to manufacturer's instructions. Data were acquired using a CyAN ADP flow cytometer. Single-fluorophore-labelled anti-mouse IgGκ /negative control (FBS) compensation particles (BD Biosciences) were used for compensation. Data were analysed offline using FlowJo (Tree Star Inc.).

Antibodies used for flow cytometry

Antibodies used in phenotyping and functional analysis of MAIT cells by flow cytometry were Anti CD3- BV421 (UCHT1), CD3-PeCy7 (SK7), CD8-PeCF594 (RPA-T8), RORγt-BV421 (Q21-559) CCR6-PE (11A9), CD45Ra-PE (HI100), CCR9-APC (112509), IL-4-PE (8D4-8), IL-6R-PE (M5) and IL-12R-APC (2.4E6) all from BD biosciences. Anti-: TCR Vα7.2-APC/Cy7 and APC (3C10), CXCR3-PE (G025H7), CD40 Ligand-PeCy7 (24-31), CD103-PE (Ber-ACT8), CD107a-PeCy5 (H4-A3), IL-18R-PE (H44), IL-5 (JES1-39D10), and IL-13 (JES10-5A2) all from BioLegend. Anti-: CD4-PerCP-Cyanine 5.5 (RPA-T4), Tbet-Pe (4B10), CD26-PE (2A6), CD73-APC (AD2), CD40L-PE (24-31), CX3CR1-PE (2A9-1), CD95-PE (DX2), CD95 Ligand/CD178-PE (NOK-1), CD11a-FITC (HI111), CD49d-PE (9F10), CD29-FITC (TS2/16), β7-Integrin-FITC (FIB504), Granzyme B-PE (GB11), IL-17-PE (eBio64CAP17), IL-22-PE

(22URTI), IFNγ-eFluor®450 (4S.B3) and TNFα-FITC and PE (MAb11) all from eBioscience. Anti-: CD8 Viogreen (BW135/80), CD161-APC or PE (191B8) IFNγ-FITC (45-15) and TCR Vα7.2-APCVio770 (REA179) all from from Miltenyi Biotec. Anti-CD69-PE (FN50) from Immunotools. Anti-: CXCR6-PE (56811), CCR5-PE (CTC5), CCR10-PE (3140305), IL-23R-PE (218213), CD18-PE (212701) and CCR7-FITC (150503) all from R&D Systems.

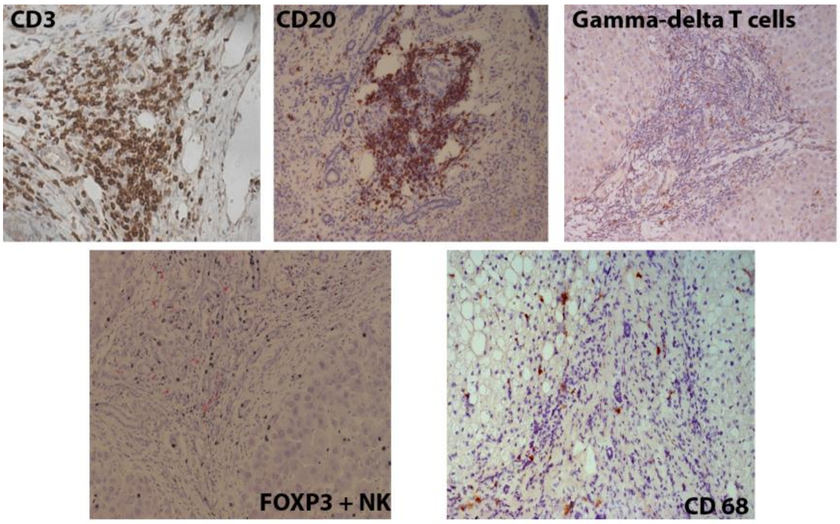

Supplementary Fig. 1. Localization of different immune cell types in the parenchyma and portal tract of human livers with chronic inflammatory liver diseases. Tissue sections from diseased human livers were stained for CD3, CD20, γδTCR, FOXP3 (brown), NKp46 (red) and CD68 to identify sites of T cell, B cell, γδ T cell, T regulatory cell and Natural Killer cell and Kupffer cell localization. Regions of parenchyma and portal tract are visible in each image illustrating the higher densities of each type of immune cell examined in the portal tract vs. parenchymal areas. Images are each representative of more than 5 donors.

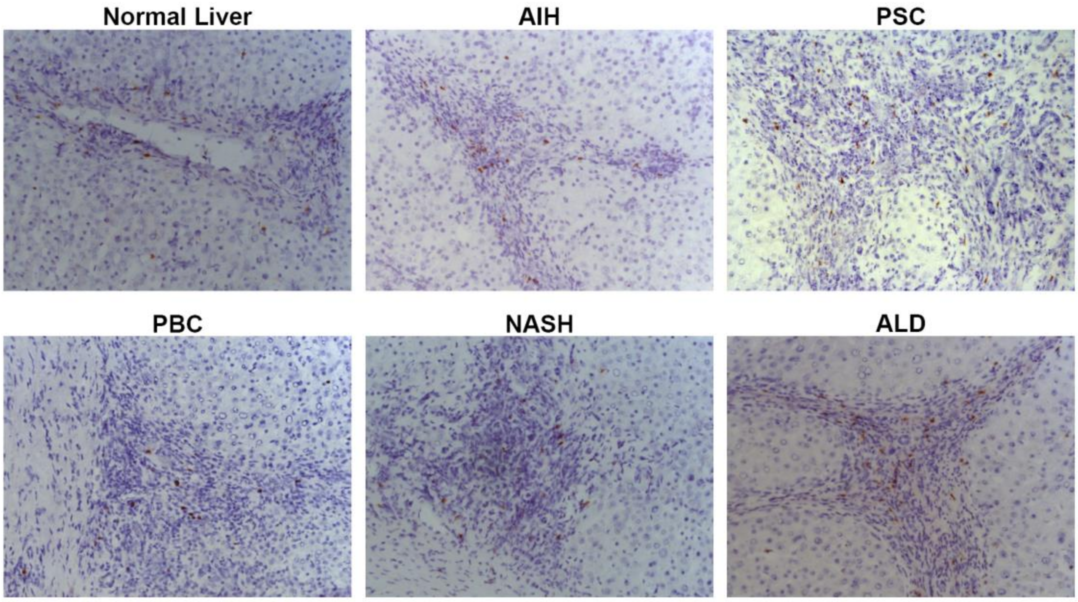

Supplementary Fig. 2. Intrahepatic localisation of Vα7.2$^+$ cells in the normal liver and chronic inflammatory liver diseases. The intrahepatic localisation of Vα7.2$^+$ cells was examined by immunohistochemistry on frozen liver tissue sections prepared from normal donor liver tissue or tissue from livers explanted for chronic diseases including: AIH, PSC, PBC, NASH and ALD. Representative images from 5 livers of staining at 20 x magnification are shown for each disease. Regions of parenchyma and portal tract are visible in each illustrating the higher densities of Vα7.2$^+$ cells (brown) in portal tract vs. parenchymal areas in the normal and diseased states.

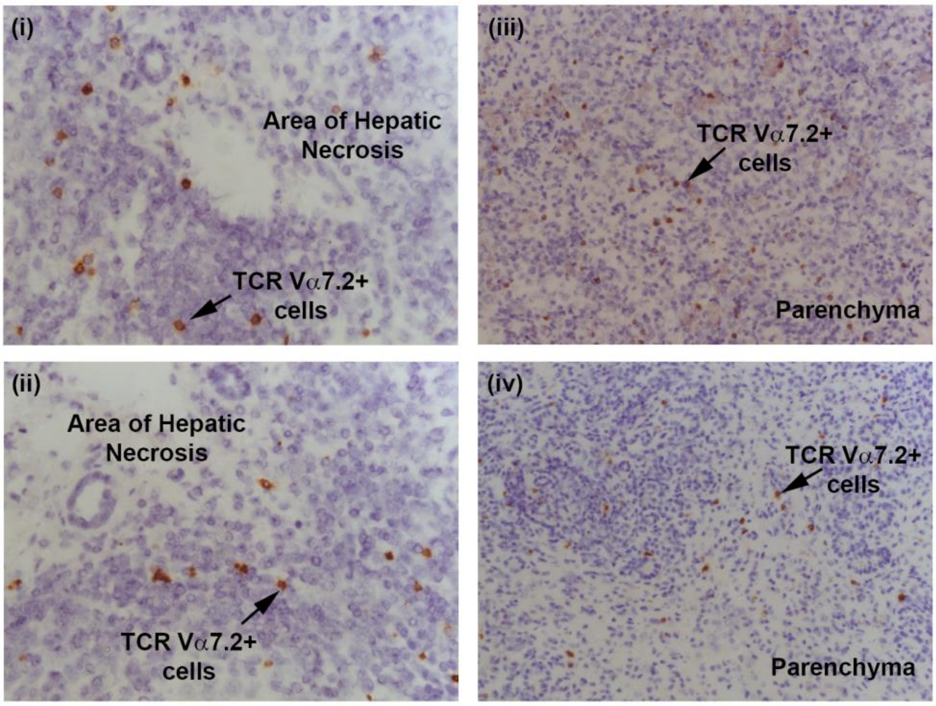

Supplementary Fig. 3. Intrahepatic localisation of Vα7.2$^+$ cells in severe acute seronegative liver failure. The intrahepatic localisation of Vα7.2$^+$ cells was examined by immunohistochemistry on frozen liver tissue sections prepared from livers explanted for severe acute seronegative liver failure. Representative images from tissues of four donors are shown. Images taken at 40 x magnification illustrate pan-acinar necrotic tissue in this disease (i and ii). Images taken at 20 x magnification illustrate the high density of Vα7.2$^+$ cells infiltration in the parenchymal tissue of this disease (iii and iv).

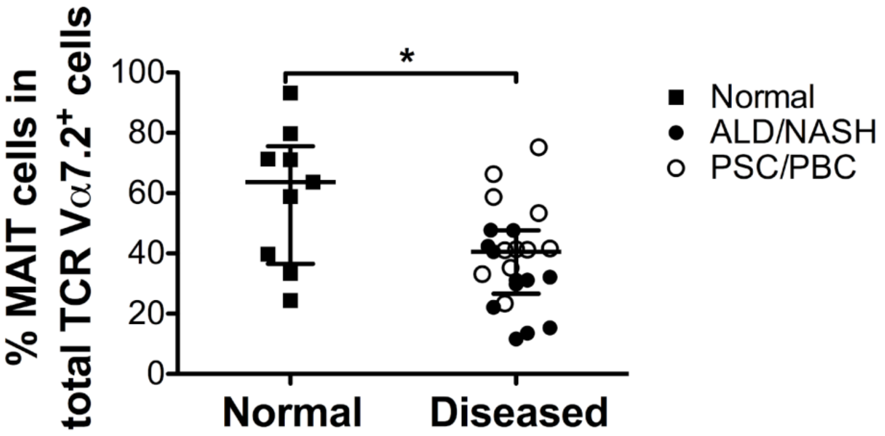

Supplementary Fig. 4. Frequencies of CD3^{+}CD161^{++} MAIT cells in total Vα7.2^{+} cells in human liver. Liver-infiltrating lymphocytes were stained with antibodies against CD3, Vα7.2, and CD161 and the frequencies of CD3^{+}CD161^{++}Vα7.2^{+} (MAIT) cells among the total Vα7.2^{+} cells determined by flow cytometry gating on the live lymphocyte population. Data are median ± interquartile range. *p <0.05 by Mann Whitney *U* test.

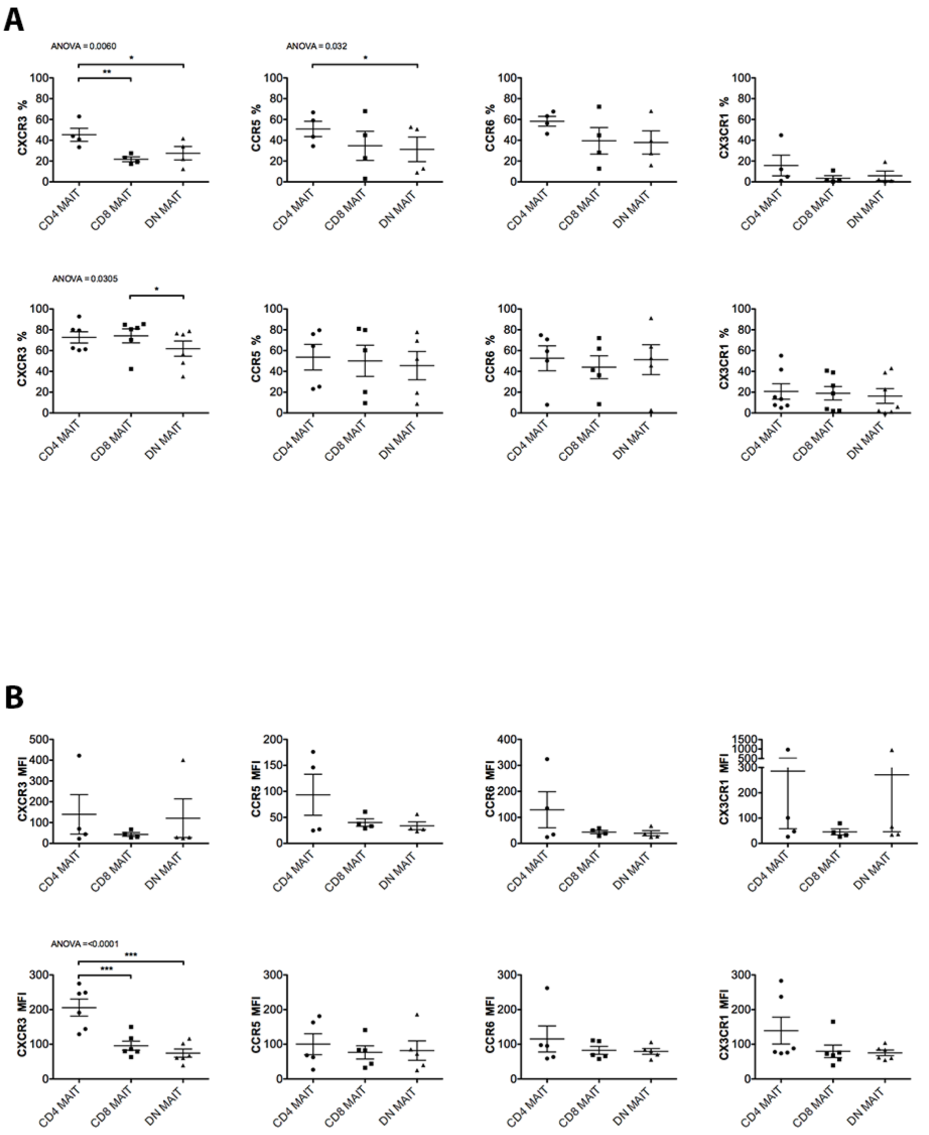

Supplementary Fig. 5. Comparison of intrahepatic CD4$^+$, CD8$^+$ and DN MAIT cell chemokine receptor expressions.

Chemokine receptor expression profiles were determined by flow cytometry gating on the CD4$^+$, CD8$^+$ and DN MAIT cell populations. Expression frequencies (A) and intensities (median fluorescence intensity (MFI) (B) were determined. Summary data are mean ± SEM. **p* <0.05; ***p* <0.01; ****p* <0.001 by Bonferroni’s Multiple Comparison post hoc test following one-way ANOVA.

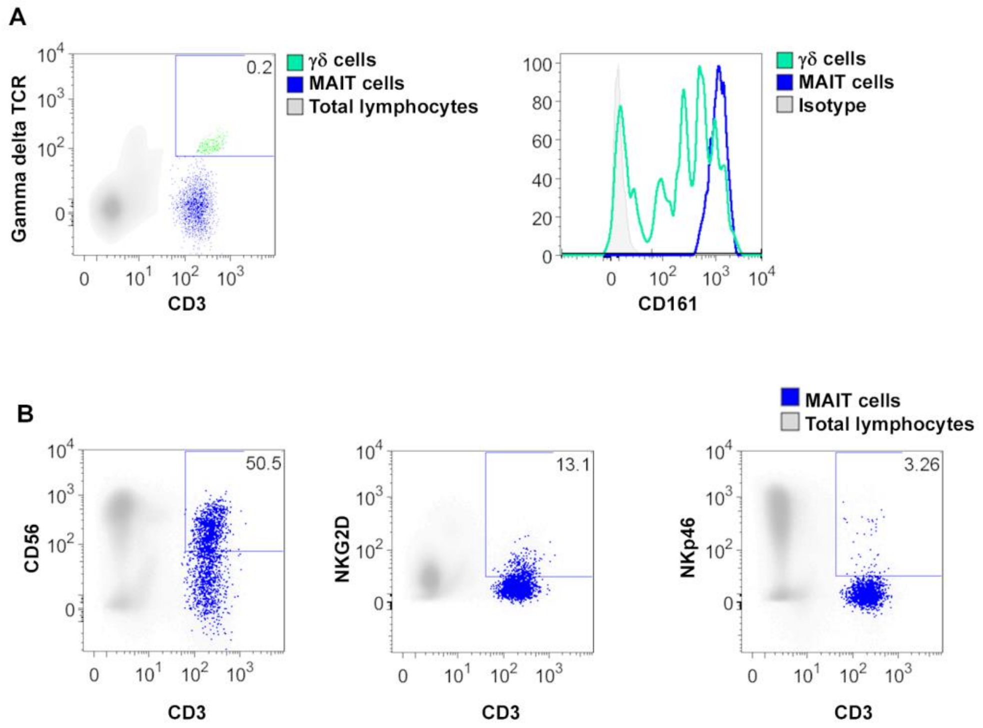

Supplementary Fig. 6. Assessment of the overlap of MAIT cells with the γδ T cells and their expression of NK cell markers. Liver infiltrating lymphocytes were stained with antibodies against MAIT markers CD3, CD161 and Va7.2 and pan γδ TCR or markers of NK cells including CD56, NKG2D and NKp46 and frequencies of γδ T cells in the MAIT population (A) or frequencies of expression of NK markers (B) by MAIT cells evaluated by flow cytometry. Data are representative of MAIT cells from three or more livers. CD161 expression by γδ T cells in relation to that of MAIT cells is also shown (A).

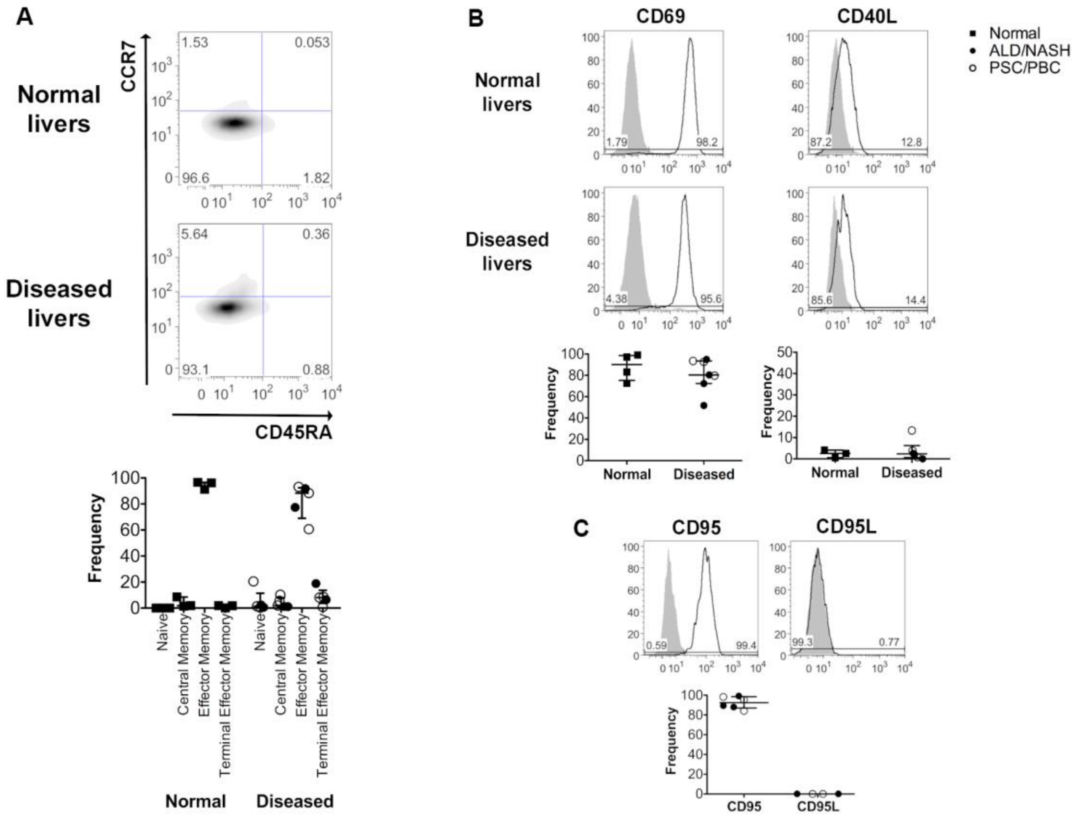

Supplementary Fig. 7. Intrahepatic MAIT cells possess an activated effector memory phenotype. Intrahepatic lymphocytes were stained with antibodies against MAIT cell markers CD3, Vα7.2, CD161 and their expression of surface receptors including CD45RA, CCR7, CD69, CD40L, CD95 and CD95L investigated by flow cytometry. (A) Representative CCR7 vs. CD45RA density plots and summary data for the proportions of naïve (CD45RA^{+}, CCR7^{+}), central memory (CD45RA^{-}CCR7^{+}), effector memory (CD45RA^{-}CCR7^{-}) and CD45RA^{+} effector memory (CD45RA^{+}CCR7^{-}) CD3^{+} MAIT cells in normal and diseased human livers. (B and C) Representative histogram overlays (marker (line), isotype (grey shading)) and summary frequency data for CD69, CD40L, CD95 and CD95L expressing CD3^{+} intrahepatic MAIT cells are shown. Data are median ± interquartile range.

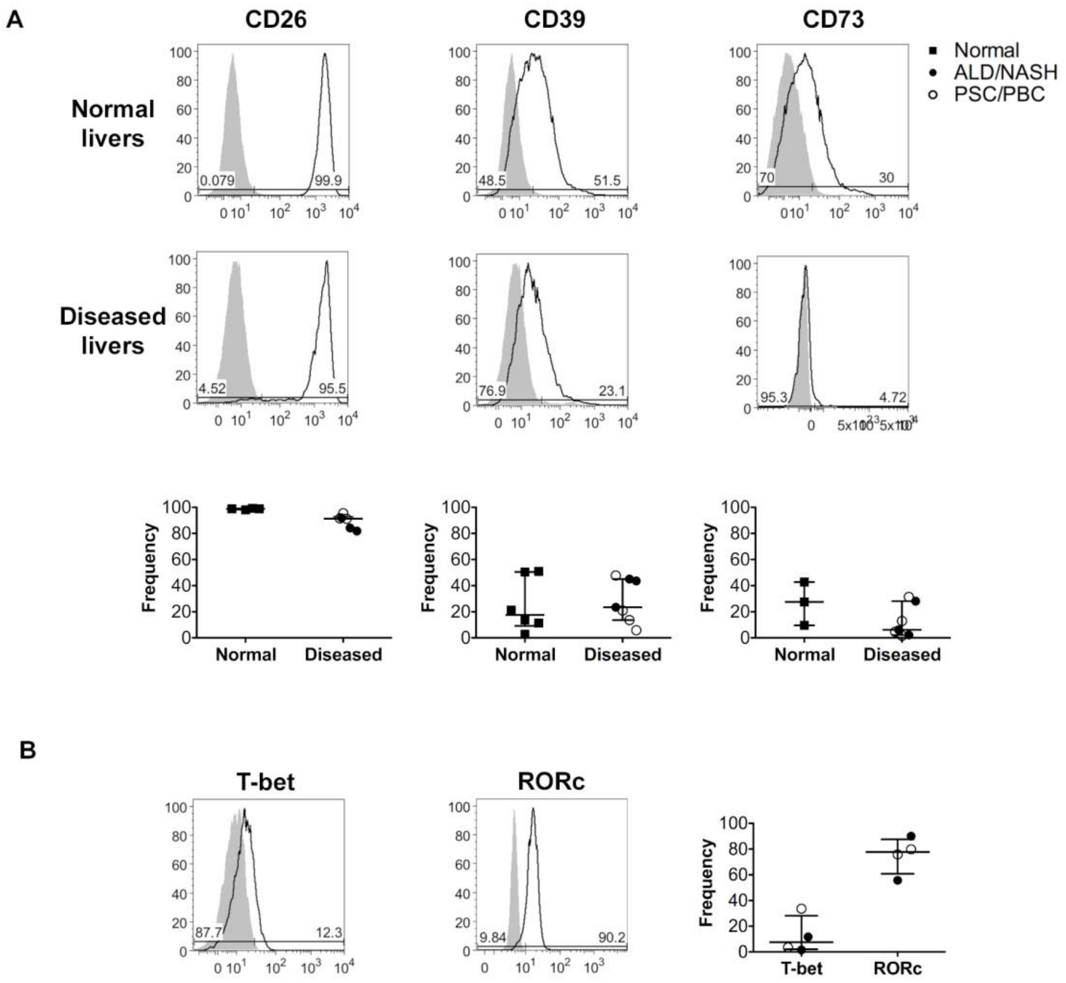

Supplementary Fig. 8. Expression of CD26, CD39 and CD73 and transcription factors by intrahepatic MAIT cells. Intrahepatic lymphocytes were stained with antibodies against MAIT cell markers CD3, Vα7.2, CD161 and their expression of surface receptors including CD26, CD39 and CD73 (A) and intracellular transcription factors Tbet and RORc (B) investigated by flow cytometry. Representative histogram overlays (marker (line), isotype (grey shading)) and summary frequency data are shown. Data are median ± interquartile range.
